# Supplementary material for: Factors influencing exclusive breast feeding among children born to HIV positive mothers attending public health facilities in western Ethiopia: Cross-sectional study
Source: PLoS One. 2022 Aug 8;17(8):e0271167. doi: 10.1371/journal.pone.0271167 (PMC9359562; doi:10.1371/journal.pone.0271167)
Supplement: Attachment — Submitted filename: Review_PONE-D-21-08205R1.docx [file pone.0271167.s003.docx]

Dear Editor

**Review of Manuscript PONE-D-21-08205**

**Comments to the authors**

I appreciate the work put in by the authors to address my earlier comments and incorporate recommendations.

Again I would like to stress the importance of having this manuscript professionally copyedited and proofread due to existing grammatical errors in the script.

The following are detailed comments and recommendations

**Results**

The authors must again review the tabular data presented as some of the frequencies and percentages are wrong.

The authors would either have to include the qualitative quotes in the results section or drop their claim of using triangulation.

I hope that the authors will consider these suggestions in improving the manuscript to address this very important topic. I wish them all the best in this endeavour.
