# Supplementary material for: Factors influencing exclusive breast feeding among children born to HIV positive mothers attending public health facilities in western Ethiopia: Cross-sectional study
Source: PLoS One. 2022 Aug 8;17(8):e0271167. doi: 10.1371/journal.pone.0271167 (PMC9359562; doi:10.1371/journal.pone.0271167)
Supplement: Attachment — Submitted filename: Response to Reviewers-II.docx [file pone.0271167.s004.docx]

**Response to Reviewers**

**Title**: Factors influencing Exclusive Breast Feeding among children born to HIV positive mothers attending public health facilities in western Ethiopia: Cross-sectional study.

**Authors**: Ejigayehu Tolessa Bultum ([ebultum@yahoo.com](mailto:ebultum@yahoo.com)

Elias Merdassa Roro ([emerdassa@gmail.com](mailto:emerdassa@gmail.com))

Tsedeke Wolde ([tsedekewolde@yahoo.com](mailto:tsedekewolde@yahoo.com))

Ilili Feyissa Regassa ([liasfeyisa2@gmail.com](mailto:liasfeyisa2@gmail.com))

**Version**: 3	**Date**: 03 April 2022

**Author’s Response to Reviewers Comments:** See over

**Editor’s Comments**

**Version: 3**

**Date:** 03 April 2022

**Title**: Factors influencing xxclusive breast feeding among children born to HIV positive mothers attending public health facilities in western Ethiopia: Cross-sectional study.

**Editor’s comment -1**

1. Again, I would like to stress the importance of having this manuscript professionally copyedited and proofread due to existing grammatical errors in the script.

**Authors Response -1**

1. Thank you for the comment. The revised manuscript has been copy edited for language usage, spelling and grammar by health professional from Australia by a native English speaker. The editor had a rich experience in editing manuscripts. The editor is currently post-doctoral fellow at the university of Queensland Mater research institute.

**Results**

**Editor’s comment -1**

1. The authors must again review the tabular data presented as some of the frequencies and percentages are wrong.
2. The authors would either have to include the qualitative quotes in the results section or drop their claim of using triangulation.

**Authors Response -1**

1. This is very relevant comment. In the revised manuscript we have revised our tables across the document. We have now revised some of the frequencies and percentages in table 2,3 & 4 in the revised manuscript. We indicated those changes using track and changes. We have now the manuscript with those changes labelled and uploaded as ‘manuscript with track change’ document.
2. We have already removed the qualitative section and dropped the triangulation claim from the manuscript.
3. We have also modified the Abstract section. This modification is also indicated in modified manuscript labeled as “manuscript with track changes”

It reads as follows:

‘In our study, only just over half of the mothers practiced EBF for the first six months. Care providers should continue to encourage mothers to practice EBF in the first six months and to disclosure of HIV status to someone close to them including their husband. Efforts should be in place to curb the risk of HIV/AIDS transmission during delivery. Continues advise for mothers to practice EBF in the first 6 months is still needed.’
